# Systematic Review and Meta-Analysis of Patients with Esophageal Cancer and Intramural Metastasis

**DOI:** 10.5761/atcs.ra.26-00153

**Published:** 2026-10-24

**Authors:** Sho Sato, Hideaki Shimada, Yuriko Takeda, Itaru Endo, Takaki Yoshikawa

**Affiliations:** 1 Department of Gastrointestinal Surgery, Yokohama City University Gastroenterological Center, Yokohama, Kanagawa, Japan; 2 Department of Surgery, Toho University School of Medicine, Tokyo, Japan; 3 Department of Biostatistics, Yokohama City University Graduate School of Medicine, Yokohama, Kanagawa, Japan; 4 Department of Gastroenterological Surgery, Yokohama City University, Yokohama, Kanagawa, Japan

**Keywords:** esophageal neoplasms, lymphatic metastasis, neoadjuvant therapy, esophagectomy, neoplasm metastasis

## Abstract

**Purpose:**

Intramural metastasis is defined as the metastatic spread from a primary tumor to the gastrointestinal tract through the intramural lymphatic system. Although several studies have demonstrated the negative impact of intramural metastasis on survival in esophageal cancer, no systematic review including a large number of patients has been conducted yet.

**Methods:**

Reports on esophageal cancer with intramural metastasis from 1990 to 2025 were collected. A pooled analysis was performed using a random-effects model to estimate the hazard ratio for overall survival in patients with intramural metastasis compared with patients without intramural metastasis.

**Results:**

Eleven studies (4494 patients) were included, in whom 315 patients (7.0%) had conventional intramural metastasis, while 57 patients had discontinuous growth. Among these patients, 4162 were diagnosed with esophageal squamous cell carcinoma. Preoperative chemotherapy was administered to 1153 patients (25.7%). The hazard ratio for overall survival was 2.22, with a 95% confidence interval of 1.81–2.72.

**Conclusions:**

Our analysis suggests that the adverse prognostic association of IM was observed across different treatment eras and remained evident in studies including patients who received neoadjuvant treatment. Future studies should explore optimal treatment strategies for patients with intramural metastasis.

## Introduction

Intramural metastasis (IM), defined as metastasis from a primary tumor to the gastrointestinal tract via the intramural lymphatic system, is the most common type of metastasis in esophageal cancer.^1)^ IM in esophageal cancer is a unique pathway that differs from hematogenous and lymphatic metastasis and is an independent prognostic factor for esophageal cancer.^2)^

IM in esophageal squamous cell carcinoma (ESCC) has been reported in 5.5% to 16.6% of cases.^2)^ The prognosis of synchronous multiple primary esophageal cancers is similar to that of a single tumor and is relatively favorable.^3,4)^ In contrast, patients with IM often have advanced lymph node metastasis and lymphatic invasion, resulting in a poor prognosis.

Although several studies have shown that IM is an important prognostic factor, the number of patients with IM has been small (mostly <50 cases), and no systematic review to date has evaluated the prognostic impact of IM in esophageal cancer. Moreover, no systematic review of IM has included articles from the Ichushi-Web database (the Japan Medical Abstracts Society, Tokyo, Japan), which includes all publications in Japan. Advances in perioperative chemotherapy have improved the prognosis of patients with locally advanced esophageal cancer; however, whether the prognosis of patients with IM has similarly improved remains unclear. Before establishing new therapeutic strategies for IM in esophageal cancer, the prognostic impact of IM should be clarified in a large patient population. Therefore, we systematically assessed the prognostic impact of IM in patients with resected esophageal cancer using a meta-analysis of the literature published in PubMed and the Ichushi-Web databases.

## Patients and Methods

This systematic review was conducted in accordance with the Preferred Reporting Items for Systematic Reviews and Meta-Analyses (PRISMA 2020) guidelines.^5)^ Ethical approval was not required for this study. The databases utilized included PubMed and the Ichushi-Web database (the Japan Medical Abstracts Society). The initial research was performed during July 2025. The literature search was updated on August 2, 2026. PubMed was searched using the following terms: (“esophageal cancer” OR “esophageal squamous cell carcinoma” OR “esophageal adenocarcinoma”) AND (“intramural metastasis” OR “intramural spread” OR “intramural lymphatic metastasis” OR “intramural deposit” OR “skip lesion” OR “gastric intramural metastasis” OR “discontinuous growth” OR “discontinuous intramural metastasis”). Ichushi-Web was searched using the corresponding English and Japanese terminology, including “esophageal cancer” and “intramural metastasis,” with case reports and conference abstracts excluded. All titles were reviewed, relevant abstracts were collected, and the screened articles were evaluated by 2 authors (S.S. and H.S.). Additionally, data extraction from each screened article was performed by the same authors.

### Eligibility criteria

The inclusion criteria were as follows: (1) observational studies providing overall survival data that compared IM vs. non-IM in the context of curative esophagectomy; (2) when multiple articles from the same institution, study group, or dataset were identified, preference was given to those with the longest follow-up duration, the largest sample size, or those written in English; and (3) for duplicate studies, the most recent and comprehensive reports were chosen. The exclusion criteria were as follows: (1) studies that did not include a comparative analysis between IM and non-IM, and (2) studies from which a hazard ratio (HR) and its 95% CI for overall survival could neither be extracted nor reliably estimated.

### Data extraction

The following data were collected: authors, publication year, country, study design, number of patients, pathological type, use of neoadjuvant chemotherapy, presence of IM, and overall survival. Additional information included the definition of IM and the variables assessed in the Cox proportional hazards regression analysis.

### Outcomes of interest and definition

The primary outcome was overall survival, expressed as the HR for patients with IM compared with those without IM. In addition, we collected the 5-year overall survival rate from each study. If not mentioned in the article, the 5-year overall survival rate was calculated using WebPlotDigitizer (https://automeris.io/) from the Kaplan–Meier curves. The HR for IM in both univariate and multivariate analyses was obtained. When HRs were not directly reported, individual patient data were reconstructed from published Kaplan–Meier curves using the method described by Guyot et al.,^6)^ and unadjusted HRs and 95% CIs were estimated using a Cox proportional hazards model. For the study by Nishimaki et al., only patients who underwent extended radical esophagectomy were included in the analysis.^7)^

### Quality assessment

Two authors (S.S. and H.S.) independently evaluated the methodological quality of the included studies using the Newcastle-Ottawa Quality Assessment Scale.^8)^ The scale evaluates 3 domains: selection (0–4 stars), comparability (0–2 stars), and outcome/exposure (0–3 stars), with a maximum score of 9 stars. Studies scoring 7 or more stars were considered high quality.

### Study protocol and registration

This systematic review was not prospectively registered in PROSPERO. However, the review was conducted according to the PRISMA 2020 statement, and the study protocol, eligibility criteria, outcomes, and statistical methods were determined before data extraction and analysis.

### Statistical analysis

For the meta-analysis, the HR and the corresponding 95% confidence interval (CI) for overall survival were extracted from each study. If Cox proportional hazards regression analysis was performed only as a univariate analysis, those data were utilized. Quantitative data were pooled using a random-effects inverse-variance–weighted meta-analysis implemented in R (version 4.5.1; R Foundation for Statistical Computing, Vienna, Austria) using the metafor package.^9)^ Heterogeneity between studies was assessed using Cochran’s Q test and quantified using the *I*^2^ statistic, which indicates the percentage of variance in a meta-analysis that is attributable to study heterogeneity. Statistical significance was defined as a 2-sided P value <0.05. We used a funnel plot and Egger’s regression test to assess publication bias. Sensitivity analysis was conducted to assess the robustness of the pooled estimates. Two sensitivity analyses were performed: (1) an analysis excluding studies for which only unadjusted HRs were available, and (2) an analysis including the study by Kmeid et al., in which discontinuous growth encompassed tumor deposits and intramural metastases and the study population consisted of patients with esophageal adenocarcinoma.^10)^

For the subgroup analysis according to treatment era, studies were classified based on the midpoint of their enrollment period rather than publication year. The year 2000 was selected as the cutoff to approximately reflect the transition toward increasing use of perioperative systemic treatment. Studies with a midpoint before 2000 were classified as the earlier-era group, whereas those with a midpoint in or after 2000 were classified as the later-era group.

## Results

### Systematic review

A flowchart of the selection process is shown in **Fig. 1**. Initially, 82 publications from PubMed and 94 from the Japan Medical Abstracts Society were identified. After eliminating duplicates, reports such as conference abstracts, case reports, book chapters, and review articles were excluded. Five additional reports were retrieved; therefore, 71 publications were assessed for eligibility. Of these, 11 publications fulfilled the inclusion and exclusion criteria. One publication identified from the Japan Medical Abstracts Society, written in Japanese, had the same results as another publication written in English; therefore, the English-language publication was included in the review.^11)^

**Fig. 1:**
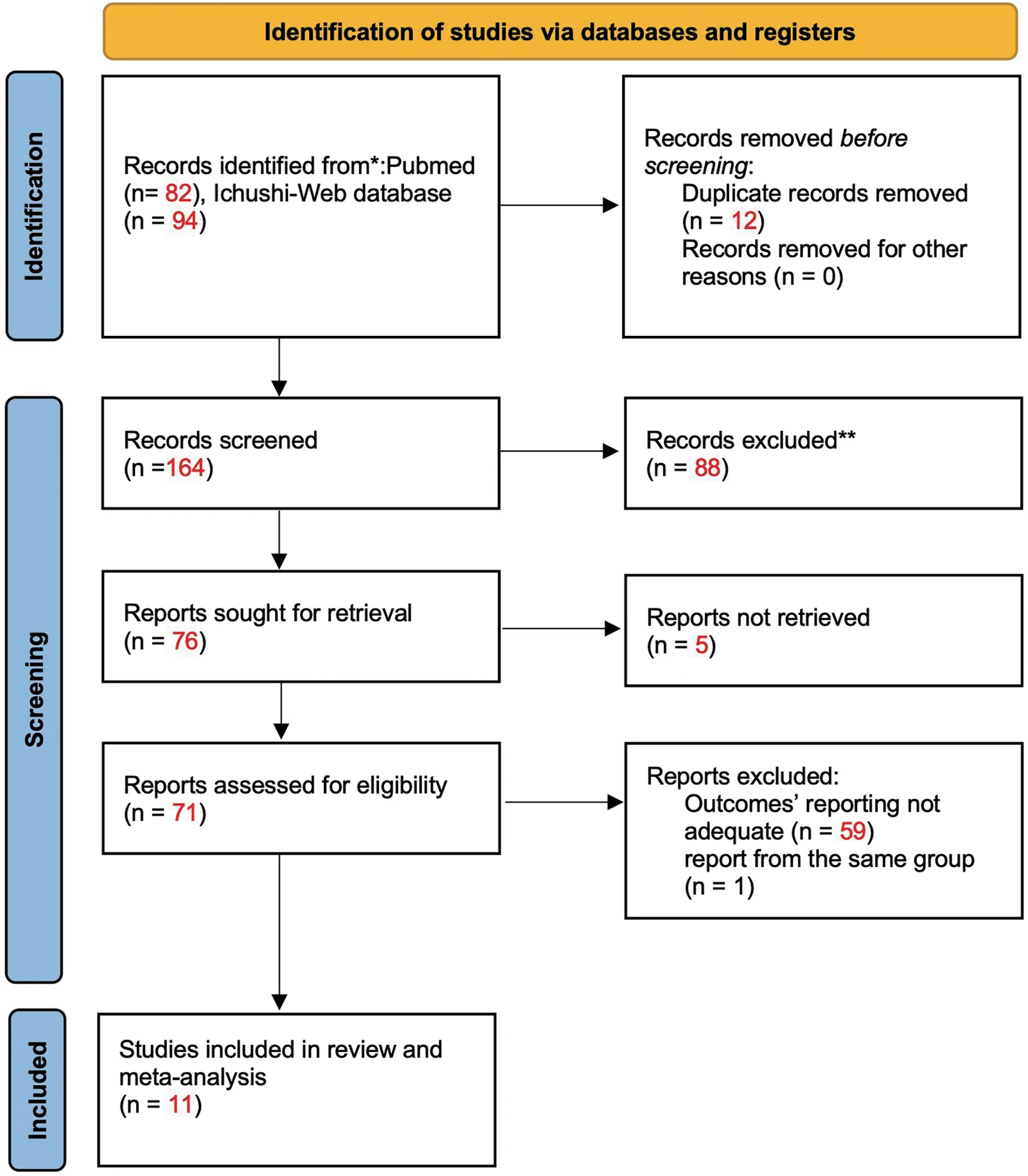
The PRISMA diagram. Eleven publications fulfilling the eligibility criteria were included in the systematic review. Ten studies evaluating conventional IM were included in the primary meta-analysis. *“esophageal cancer” OR “esophageal squamous cell carcinoma” OR “esophageal adenocarcinoma”) AND (“intramural metastasis” OR “intramural spread” OR “intramural lymphatic metastasis” OR “intramural deposit” OR “skip lesion” OR “gastric intramural metastasis” OR “discontinuous growth” OR “discontinuous intramural metastasis”. Ichushi-Web was searched using the corresponding English and Japanese terminology, including “esophageal cancer” and “intramural metastasis,” with case reports and conference abstracts excluded. **Records excluded by title and abstract screening due to publication type (e.g., non-esophageal cancer, conference abstracts, case reports, book chapters, and review article, written in non-English/Japanese). PRISMA, Preferred Reporting Items for Systematic Reviews and Meta-Analyses

A summary of the reports is shown in **Table 1**.^1,2,7,10–17)^ The definition of IM is described in **Supplementary Table 1**. The study period ranged from 1970 to 2022. A total of 4494 patients were included in the systematic review. Of these, 315 patients (7.0%) had conventional IM, while 57 patients had discontinuous growth as defined by Kmeid et al. Overall, 4162 patients (92.6%) were diagnosed with ESCC. Preoperative chemotherapy was administered to 1153 patients (25.7%), and preoperative chemoradiotherapy or radiotherapy was administered to 247 patients (5.5%); however, the proportion and regimens of preoperative treatment differed between studies. Four studies did not include patients undergoing preoperative treatment, whereas the others included patients who received preoperative radiotherapy or chemoradiotherapy, fluorouracil plus cisplatin therapy, or docetaxel plus fluorouracil plus cisplatin therapy. The 5-year overall survival rate of the IM group was lower than that of the non-IM group (0%–18.1% in the IM group; 27.2%–62.6% in the non-IM group).

**Table 1:** Summary of the reports showing clinicopathological characteristics of patients with esophageal cancer

Author, Year [Ref]	Study period	Study design	Pathology	Group	No. Pts	Neoadjuvant treatment	5y-OS	HR (95% CI)
Endo T, 1992^12)^	1980–1989	Ret	ESCC 410	IM	52	RT 93 (22.7%)	16.4%	2.06 (1.35–3.14)
				Non-IM	358	NAC 73 (17.8%)	44.1%	
Kuwano H, 1994^13)^	1970–1992	Ret	ESCC 155/OTH 12	IM	24	None	5.8%*	2.04 (1.00–4.14)
				Non-IM	143	None	27.2%*	
Nishimaki T, 1996^7)^	1982–1993	Ret	ESCC 155	IM	24	None	0%	3.95 (2.30–6.77)
				Non-IM	131	None	45.5%	
Yuasa N, 2004^14)^	1980–2020	Ret	ESCC 212	IM	23	None	9%	2.04 (1.17–3.56)
				Non-IM	189	None	42%	
Tanabe T, 2007^15)^	1992–2003	Ret	ESCC 171	IM	20	None	–	1.16 (0.61–2.20)
				Non-IM	151	None	–	
Ichikawa H, 2013^16)^	1995–2010	Pro	ESCC 267/BAS 16/ AC 21/ OTH 11	IM	26	NAC 74 (23.5)	–	2.84 (1.74–4.62)
				Non-IM	289		–	
Okamura A, 2019^2)^	2005–2016	Ret	ESCC 918	IM	46	NAC 22 (50%)	17.4%*	2.04 (1.41–2.96)
						CRT 1 (2.2)		
				Non-IM	872	NAC 334 (38.3%)	62.6%*	
						CRT 66 (7.6)		
Ushimaru Y, 2023^1)^	2010–2016	Ret	ESCC 597	IM	23	NAC 21 (91%)	14.2%	2.02 (1.25–3.27)
				Non-IM	574	NAC 486 (84%)	58.2%	
Murakami T, 2024^11)^	2012–2020	Ret	ESCC 194	IM	18	RT 87 (45%)	–	1.53 (0.80–2.93)
				Non-IM	176	NAC 10 (5%)	–	
Yusupbekov A, 2024^17)^	2001–2021	Ret	ESCC 1083	IM	59	NAC 6 (10.2%)	12.8%*	2.56 (1.92–3.42)
				Non-IM	1024	NAC 6 (6.5%)	33.1%*	
Kmeid M, 2024^10)^	2003–2022	Ret	AC 272	DG	57	NAC 35 (61.4%)	18.1%*	1.74 (1.09–2.77)
				Non-DG	213	NAC 86 (40.4)	57.7%*	

*Calculated by WebPlotDigitizer (URL: https://automeris.io/).

CI, confidence interval; ESCC, esophageal squamous cell carcinoma; HR, hazard ratio; IM, intramural metastasis; OTH, others; BAS, basaloid; AC, adenocarcinoma; NAC, neoadjuvant chemotherapy; CRT, chemoradiotherapy; RT, radiotherapy; DG discontinuous growth

### Meta-analysis primary outcomes

Among the 11 studies, the study by Kmeid et al. was broader than the conventional definition of IM. In particular, discontinuous growth in their study encompassed tumor deposits, intramural deposits, and submucosal deposits and therefore may not be directly comparable with conventional IM. Therefore, the primary meta-analysis was restricted to 10 studies evaluating conventional IM, and the study by Kmeid et al. was excluded from the primary analysis (**Table 2**). The pooled HR was estimated to compare IM with the non-IM group (**Fig. 2**). The HR was 2.22 (95% CI, 1.81–2.72). No significant heterogeneity was observed (Cochran’s Q = 4.61, df = 9, P = 0.867, *I*^2^ = 0%). Egger’s regression test did not indicate significant funnel plot asymmetry (P = 0.238; **Fig. 3**). Sensitivity analyses excluding studies for which only unadjusted HRs were available and including the Kmeid study yielded pooled HRs of 1.89 (95% CI, 1.56–2.29; **Supplementary Fig. 1**) and 2.17 (95% CI, 1.80–2.62; **Supplementary Fig. 2**), respectively. The adverse prognostic association of IM was observed in both treatment eras. The adverse prognostic association of IM was observed in both treatment eras. The pooled HRs were 2.13 (95% CI, 0.97–4.71) for studies with a study-period midpoint before 2000 and 2.26 (95% CI, 1.86–2.74) for those with a midpoint in or after 2000. There was no statistically significant difference between the 2 subgroups (P for subgroup difference = 0.8287) (**Supplementary Fig. 3**). Furthermore, the pooled HRs for IM were 2.13 (95% CI, 0.97–4.78) in studies without neoadjuvant treatment and 2.25 (95% CI, 1.86–2.70) in studies, including patients who received neoadjuvant treatment. There was no statistically significant difference between the 2 subgroups (P for subgroup difference = 0.842). The magnitude of the adverse prognostic association of IM was similar between the 2 groups, although the 95% CI crossed unity in the subgroup of studies without neoadjuvant treatment (**Supplementary Fig. 4**).

**Table 2:** Covariates included in survival analyses

Author, Year [Ref]	Covariates in multivariate analysis
Endo T, 1992^12)^	pT, pN, Location of the tumor, Size, Lymphatic invasion, Venous invasion, Cellular atypism, Structural atypism, Infiltration, intraepithelial spread, Gross type, Intramural metastasis
Kuwano H, 1994^13)^	pT, Size, Intramural metastasis
Yuasa N, 2004^14)^	pT, pN, Lymphatic invasion, Intramural metastasis
Nishimaki T, 1996^7)^	Intramural metastasis*
Tanabe T, 2007^15)^	pT, Growth pattern, Venous invasion, Number of metastatic nodes, Extranodal spreading, Intramural metastasis
Ichikawa H, 2013^16)^	Intramural metastasis*
Okamura A, 2019^2)^	pT, pN, pM, Age, BMI, Sex, Preoperative treatment, Curability, Intramural metastasis
Ushimaru Y, 2023^1)^	pT, pN, pM, Histological differentiation, Intramural metastasis
Murakami T, 2024^11)^	pT, pN, Preoperative treatment, Lymphatic invasion, Venous invasion, PD-L1 stroma, PD-L1 tumor, PD-L1 stroma + tumor, Intramural metastasis
Yusupbekov A, 2024^17)^	Intramural metastasis*
Kmeid M, 2024^10)^	Neoadjuvant treatment, Size, Lymphatic invasion, Margin, pT, pN, Metastasis, Surgery year, Discontinuous growth

*This study evaluated the hazard ratio in univariate analysis or the data of multivariate analysis was not adequate.

**Fig. 2:**
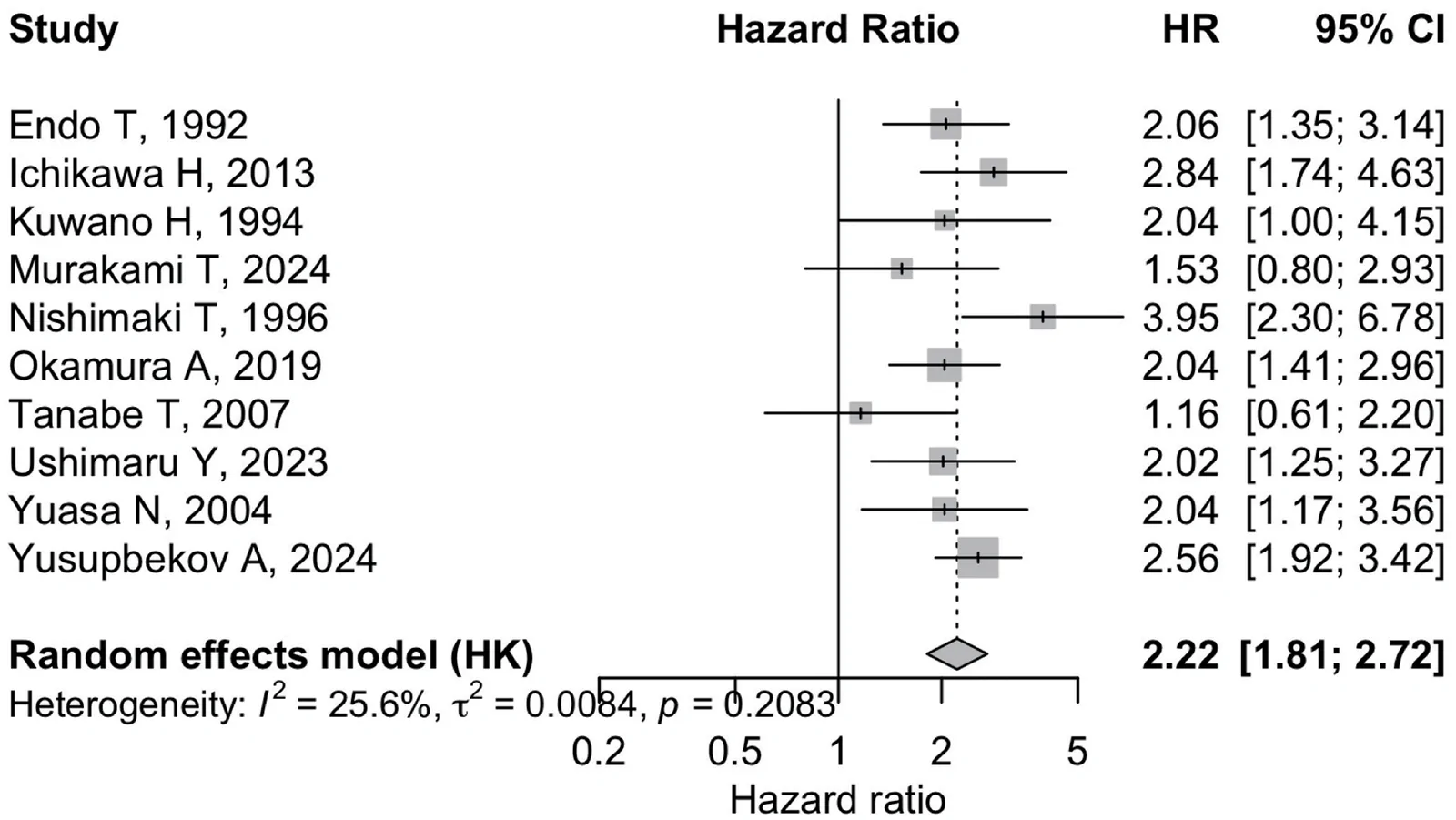
Forest plot of hazard ratio in esophageal cancer with intramural metastasis. The pooled estimates were calculated using a random-effects model to account for between-study heterogeneity. HR, hazard ratio; CI, confidence interval

**Fig. 3:**
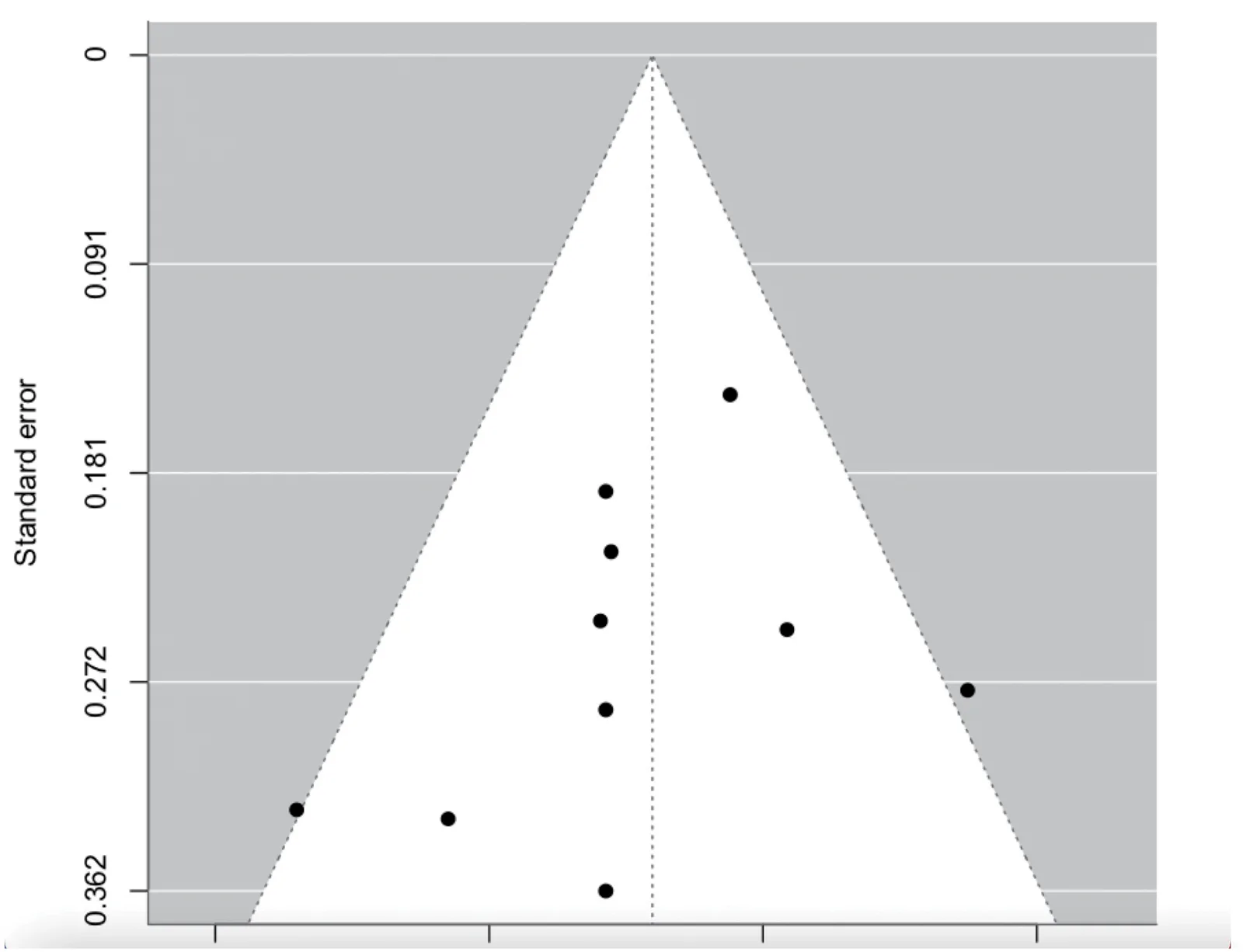
Funnel plot for the assessment of publication bias. The funnel plot shows the log hazard ratios of individual studies plotted against their standard errors. Egger’s regression test did not indicate significant funnel plot asymmetry (P = 0.238).

### Quality assessment of included studies

The Newcastle-Ottawa Quality Assessment Scale scores of the included studies ranged from 5 to 8, with 4 studies considered high quality (scores ≥7) (**Table 3**). The main limitations were related to comparability and the adequacy of follow-up.

**Table 3:** Quality assessment of included studies using the Newcastle–Ottawa scale

Author, Year [Ref]	Selection (0–4)	Comparability (0–2)	Outcome/Exposure (0–3)	Total (0–9)
Endo T, 1992^12)^	3	1	1	5
Kuwano H, 1994^13)^	4	1	1	6
Nishimaki T, 1996^7)^	4	0	2	6
Yuasa N, 2004^14)^	4	1	2	7
Tanabe T, 2007^15)^	3	1	2	6
Ichikawa H, 2013^16)^	3	1	2	6
Okamura A, 2019^2)^	4	2	2	8
Ushimaru Y, 2023^1)^	4	1	2	7
Murakami T, 2024^11)^	3	1	2	6
Yusupbekov A, 2024^17)^	4	0	2	6
Kmeid M, 2024^10)^	4	1	2	7

## Discussion

We conducted a meta-analysis to evaluate the prognostic impact of IM in patients with resected esophageal cancer using 11 reports from which sufficient data were extracted from PubMed and the Ichushi-Web database. Despite substantial changes in treatment strategies over the study period, IM remained consistently associated with poor overall survival. To the best of our knowledge, this is the first meta-analysis to report the prognostic impact of IM covering both PubMed and the Ichushi-Web database.

The principal strength of the present meta-analysis is that it quantitatively evaluated the prognostic impact of IM across studies spanning approximately 5 decades, during which treatment strategies for esophageal cancer have changed substantially. Although the poor prognosis associated with IM has been reported in individual studies, the present study demonstrated a consistently adverse association between IM and overall survival across different treatment eras. Notably, the magnitude of this association was similar between studies with a study-period midpoint before 2000 and those with a midpoint in or after 2000, with no significant subgroup difference. Similarly, the pooled HRs were comparable between studies without neoadjuvant treatment and those including patients who received neoadjuvant treatment. These findings suggest that IM remains an important marker of poor prognosis despite advances in perioperative treatment, although the limited number of studies and the heterogeneity of treatment strategies warrant cautious interpretation.^18–21)^

Limited evidence is available regarding the interaction between IM and tumor immune microenvironment. Murakami et al. reported that PD-L1 expression, rather than IM, remained an independent prognostic factor in multivariable analysis.^11)^ Further studies are needed to clarify the biological relationship between IM and antitumor immunity.

Because IM is associated with aggressive lymphatic invasion, extranodal spread in ESCC may correlate with IM. Tanabe et al. evaluated the prognostic impact of extranodal spread in ESCC and demonstrated that patients with extranodal spread positivity had significantly poorer survival than those without extranodal spread. In addition, patients with extranodal spread exhibited IM and a higher number of metastatic nodes more frequently than those without extranodal spread. Because IM may be confounded by extranodal spread and the number of metastatic lymph nodes, the HR for IM was attenuated (HR 1.16; 95% CI, 0.61–2.1).^15)^ These findings suggest that the adverse prognostic effect attributed to IM may, at least in part, reflect extensive lymphatic dissemination and nodal tumor burden.

Among the 11 studies, only one evaluated the impact of IM in patients with esophageal adenocarcinoma. Kmeid et al. defined “discontinuous growth” as a tumor with either IM or tumor deposits.^10)^ Tumor deposits are defined as discrete, viable tumor foci located in the peritumoral adventitial tissue or perinodal fat and are recognized as a prognostic factor in colorectal cancer.^22)^ Kmeid study did not distinguish between tumor deposits and IM, which represents a limitation of the present systematic review. However, a sensitivity analysis including the Kmeid study yielded a pooled HR of 2.17 (95% CI, 1.80–2.62), which was comparable to that of the primary analysis excluding the Kmeid study (HR, 2.22; 95% CI, 1.81–2.72). These findings suggest that inclusion of the Kmeid study did not materially alter the pooled estimate.

Treatment strategies for esophageal cancer with IM are challenging. Hokamura et al. reported poor long-term outcomes in patients with IM despite preoperative chemotherapy, with a 5-year overall survival rate of only 20%.^23)^ These findings highlight the need for improved treatment strategies for this high-risk population. Given the poor prognosis associated with IM, optimization of perioperative treatment remains an important unmet need. Although recent clinical trials have demonstrated promising activity of immune-checkpoint inhibitors in esophageal cancer, whether patients with IM derive particular benefit from immunotherapy remains unknown.^24–27)^ Thus, the potential role of immunotherapy in patients with IM is currently hypothesis-generating and requires further investigation. In the present meta-analysis, IM remained associated with poor prognosis in studies including patients who received neoadjuvant treatment. Therefore, the optimal perioperative treatment strategy for this high-risk population remains to be established.

This study has several limitations. First, the definition of IM differed between studies. Some studies diagnosed IM before surgical resection, while others diagnosed it using resected specimens. Second, three of the included studies reported HRs derived from univariate analyses, which may have resulted in residual confounding and affected the pooled estimates. Third, there is a possibility of missing studies during screening. Although we searched PubMed and the Ichushi-Web database, which cover most domestic and international studies, we cannot deny the presence of missing articles. Fourth, although Egger’s regression test did not indicate significant funnel plot asymmetry, the small number of included studies limits the statistical power of tests for publication bias. Therefore, the possibility of publication bias cannot be completely excluded. Although the direction and magnitude of the association remained consistent in the sensitivity analyses, the results should be interpreted with caution, and further large-scale studies are warranted to confirm the prognostic significance of IM.

In conclusion, IM was associated with poor survival outcomes in patients with esophageal cancer. Given the unfavorable prognosis of these patients, further studies are warranted to explore whether novel perioperative treatment strategies, including those incorporating immune-checkpoint inhibitors, may improve clinical outcomes.

## Supplementary Materials

Supplementary Table 1

Supplementary Figs. 1–4

## Declarations

### Data availability

Data generated at a central, large-scale facility are available upon reasonable request from the corresponding author.
