# Supplementary material for: Systematic Review and Meta-Analysis of Patients with Esophageal Cancer and Intramural Metastasis
Source: Ann Thorac Cardiovasc Surg. 2026 Oct 24;32(1):26-00153. doi: 10.5761/atcs.ra.26-00153 (PMC13711103; doi:10.5761/atcs.ra.26-00153)
Supplement: Supplementary file 1 [file atcs-32-1-26-00153-s001.pdf]

Supplementary Table 1.

Definition of Intramural Metastasis

Author, Year [Ref]	Definition of intramural metastasis
Endo T, 1992 [12]	Not mentioned
Kuwano H, 1994 [13]	(1) clearly separated from the primary tumor, (2) located within the esophageal wall, (3) not located within a vessel lumen but rather observed to invade the esophageal stroma, (4) not accompanied by an intraepithelial cancerous lesion
Nishimaki T, 1996 [7]	(1) clearly separated from the primary tumor; (2) located in the esophageal or stomach wall; (3) having a gross appearance of a submucosal tumor without intra- epithelial cancer extension; (4) having the same histologic type as the primary tumor; and (5) lacking any evidence of intravascular growth.
Yuasa N, 2004 [14]	(1) clearly separated from the primary tumor, (2) located in the esophageal or gastric wall, (3) not surrounded by endothelium, and (4) not accompanied by extension of intraepithelial cancer.
Tanabe T, 2007 [15]	Not mentioned

Ichikawa H, 2013 [16]	Not mentioned
Okamura A, 2019 [2]	Defined as metastasis that was clearly separate from the primary tumor and located within the esophageal or gastric wall. In cases after neoadjuvant therapy, we diagnosed the isolated tumor nests which lacked apparent therapeutic changes as IM.
Ushimaru Y, 2023 [1]	(1) clearly separated from the primary tumor; (2) located in the esophageal wall; (3) having a gross appearance of a submucosal tumor without intraepithelial cancer extension; (4) having the same histologic type as the primary tumor in the cases with the histological examination; and (5) lacking any evidence of intravascular growth.<br>In the case that endoscopic biopsy did not lead to a diagnosis, the IM diagnosis was based on comprehensive findings, including CT, positron emission tomography (PET)-CT, and postoperative pathology.
Murakami T, 2024 [11]	Not mentioned
Yusupbekov A, 2024 [17]	(1) the clear nodular nature of the metastatic node, (2) submucosal localization in the wall of the esophagus or stomach without an inflammatory reaction around the node, and (3) undetectable mucosal involvement in the metastatic node

Kmeid M, 2024 [10]	Discontinuous growth was classified into 3 types: tumor deposits, intramural deposits, and submucosal deposits. (1) Tumor deposits were defines as discrete, viable tumor foci, that were separate from the primary carcinoma, and present in the peritumoral adventitial adipose tissue or perinodal fat, (2) intramural deposits and submucosal deposits were defined as tumor foci that were clearly discontinuous from the primary tumor (2 mm), irrespective of size, not associated with vascular or neural structures, and identified in the muscularis wall propria of the esophageal or in the submucosa, respectively.
