# Supplementary material for: Systematic Review and Meta-Analysis of Patients with Esophageal Cancer and Intramural Metastasis
Source: Ann Thorac Cardiovasc Surg. 2026 Oct 24;32(1):26-00153. doi: 10.5761/atcs.ra.26-00153 (PMC13711103; doi:10.5761/atcs.ra.26-00153)

Supplementary Figure 1

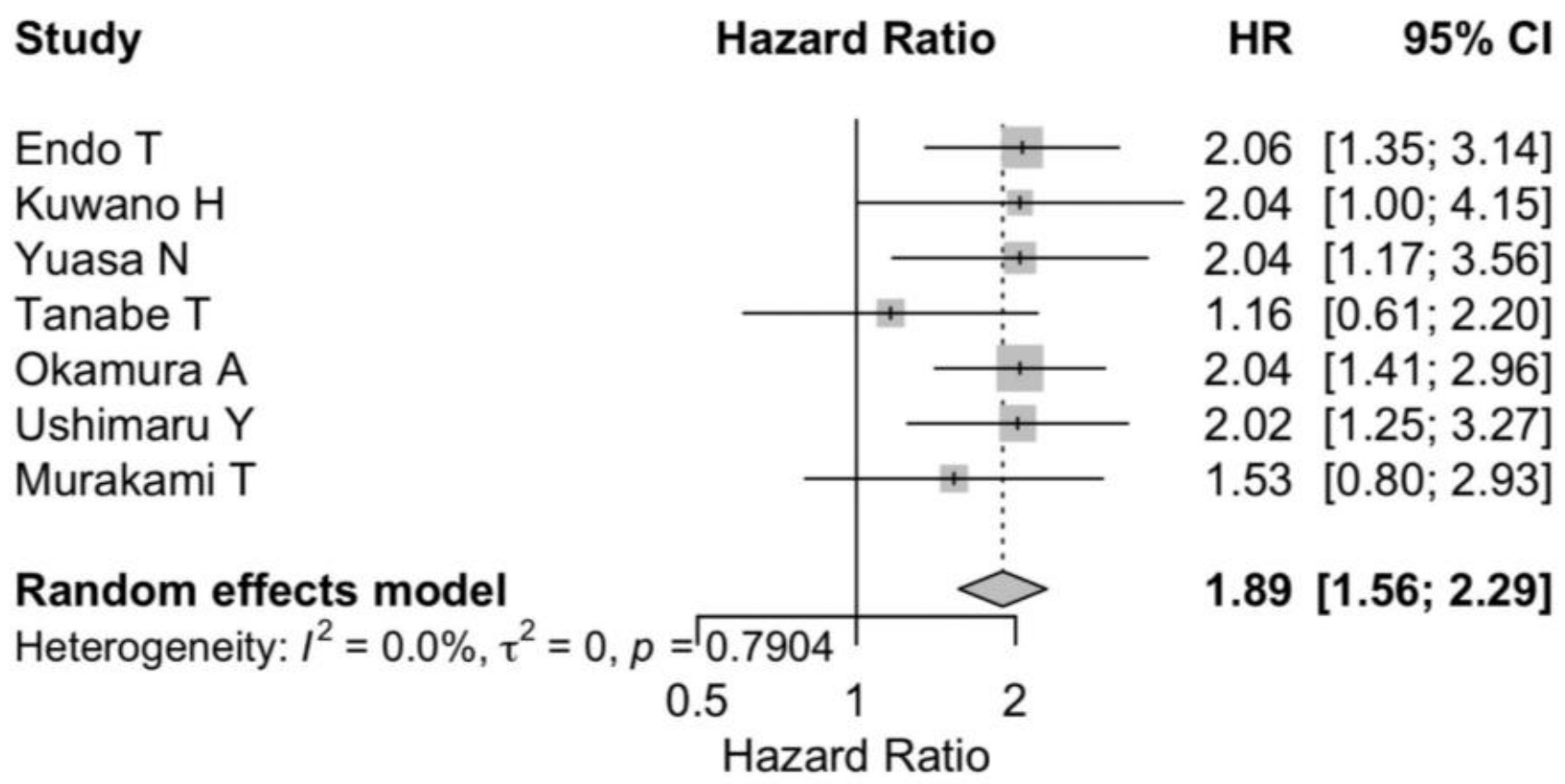

Supplementary Figure 2

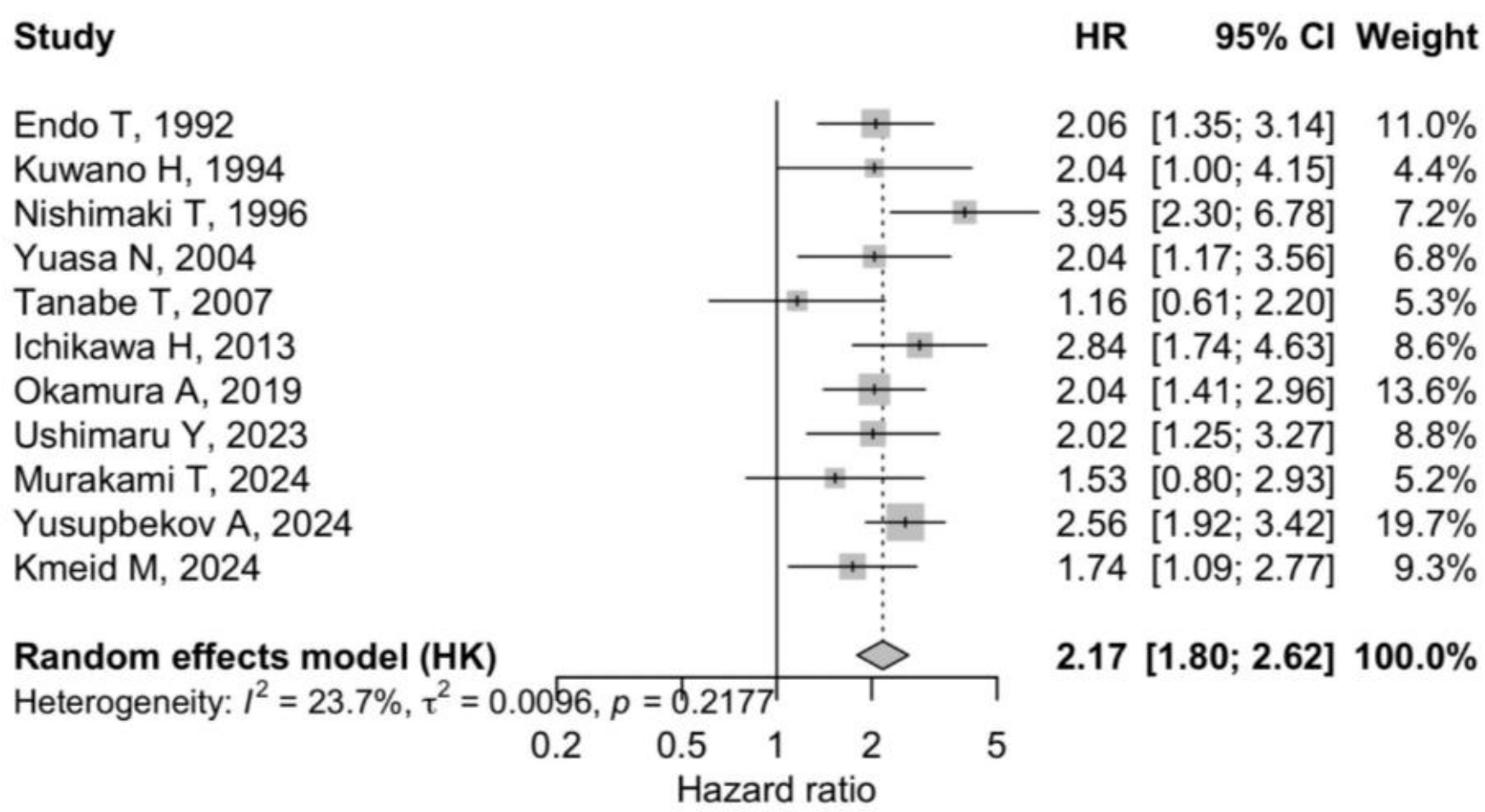

Supplementary Figure 3

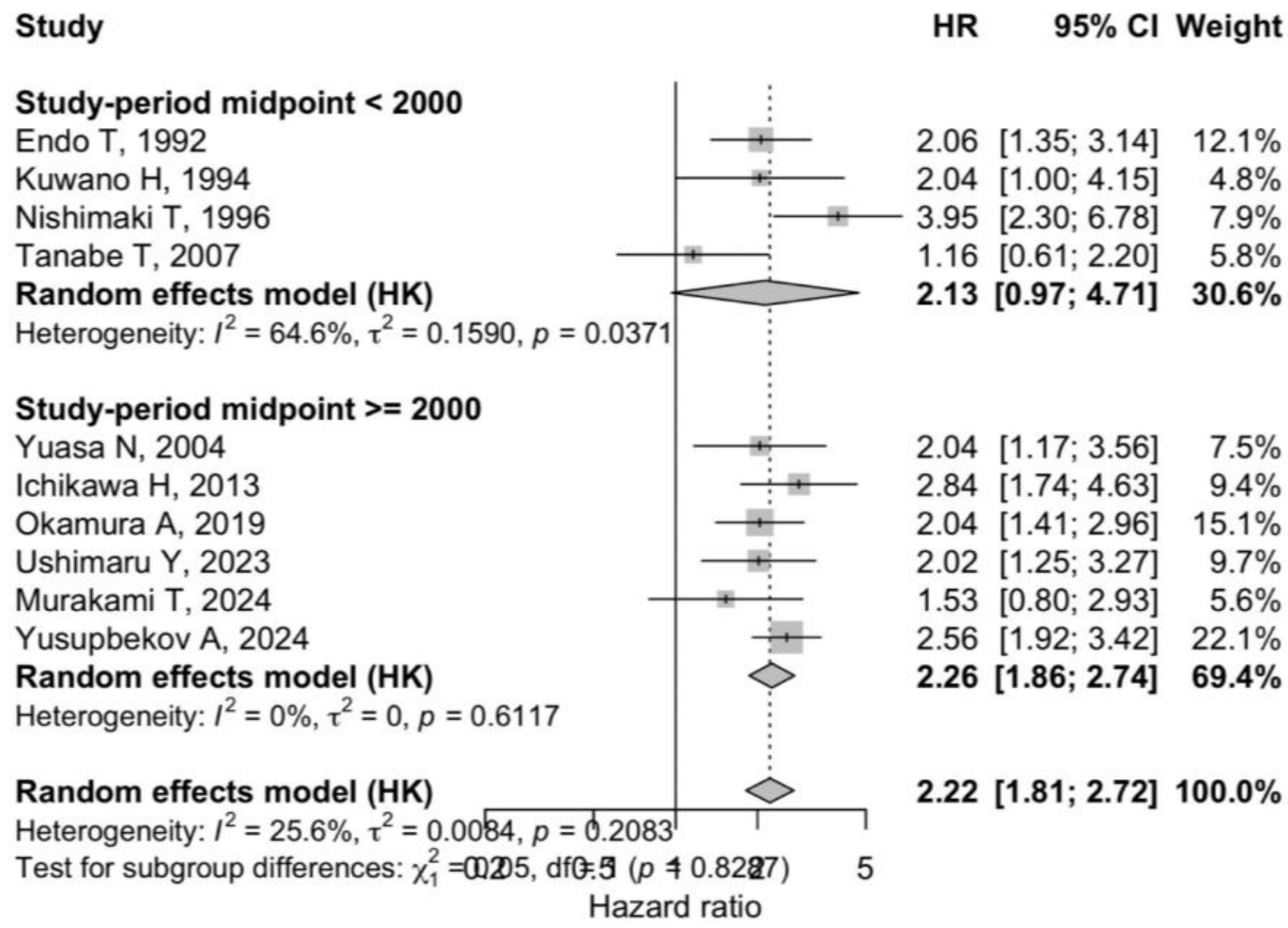

Supplementary Figure 4

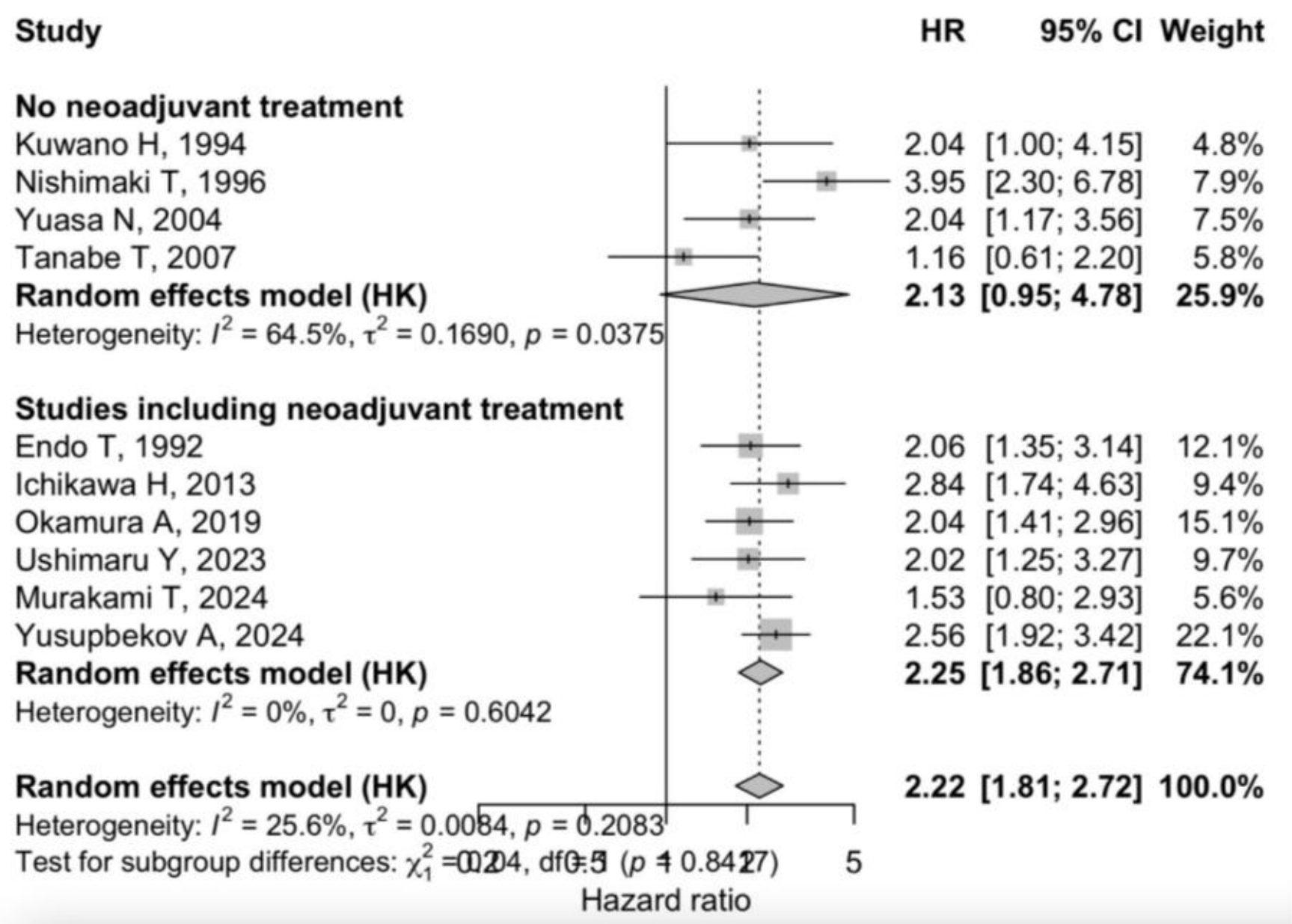

Supplement: Supplementary file 2 [file atcs-32-1-26-00153-s002.pdf]
